# Energy Landscape and Electronic Properties of Yttrium Oxysulfide (Y_2_O_2_S)

**DOI:** 10.3390/ma19184018

**Published:** 2026-09-21

**Authors:** Dragana Jordanov, Jelena Zagorac, Johann Christian Schön, Klaus Doll, Dejan Zagorac

**Affiliations:** 1 Materials Science Laboratory, Institute of Nuclear Sciences Vinča, University of Belgrade, 11351 Belgrade, Serbia; 2 Max Planck Institute for Solid State Research, 70569 Stuttgart, Germany; 3 Institute of Theoretical Chemistry, University of Stuttgart, 70569 Stuttgart, Germany

**Keywords:** yttrium oxysulfide, Y_2_O_2_S, rare-earth elements (REEs), wide-bandgap semiconductors, DFT, polymorphs

## Abstract

This study investigates the electronic properties and crystalline phases of yttrium oxysulfide (Y_2_O_2_S) using theoretical computational methods. A crystal structure prediction was employed to explore the compound’s energy landscape. A global optimization using empirical potentials was performed, followed by a local optimization via ab initio calculations using the generalized gradient approximation (GGA), the local density approximation (LDA), and the hybrid B3LYP functional. The computational results confirm the stability of the alpha phase, whose structure aligns with the experimentally observed trigonal modification. Furthermore, four previously unknown metastable polymorphic phases, *β*-, *γ*-, *δ*-, and *ε*-Y_2_O_2_S structures, were identified. An examination of the electronic structure reveals that Y_2_O_2_S, a wide-bandgap semiconductor, possesses an indirect energy gap. The results obtained show good agreement with the available literature. A further analysis of the structure–property relationship of the predicted Y_2_O_2_S polymorphs indicates the possibility of an indirect-to-direct bandgap transition, which could have significant applications in optoelectronics.

## 1. Introduction

Materials containing rare-earth elements (REEs) exhibit chemical and physical properties of great interest to modern industrial processes with applications in many fields, including solar energy, wind turbines, batteries for electric vehicles and mobile phones, and color display devices. These materials are realized as glasses, metal alloys, or ceramics, where the REE is present as a constituent material, such as in the ceramic yttrium oxide [1,2], and/or as a dopant. The core motivation for selecting specific dopants in Y_2_O_2_S relies on a strategic balance of structural symmetry compatibility, low phonon energy exploitation, a wide bandgap (WBG), and intentional defect engineering.

In this study, the focus is on yttrium oxysulfide (Y_2_O_2_S), which has received considerable attention as a phosphorescent host material and is widely used in the luminescent materials industry [3,4]. When doped with Co^2+^, Mn^2+,^ and/or rare-earth cations, such as europium, terbium, or erbium, in various concentrations, yttrium oxysulfide exhibits luminescence and can be used as a cathodoluminescent material [5,6,7,8]. Due to the special electronic structure of Eu^3+^, it has been a preferred dopant: Eu-activated Y_2_O_2_S emits bright red light under cathode-ray excitation, and it has been widely used as an important material for color display devices [8,9]. In particular, when co-doped with Mg^2+^ and Ti^4+^, it is employed as a long-afterglow (persistent luminescence) material with excellent stability and high-quality performance [10,11,12,13,14]. For example, the Y_2_O_2_S:Eu,Mg,Ti nanotube arrays, synthesized via the sol–gel template method, are used in biochemistry and medical devices due to their highly ordered, integrated structure [15].

Nevertheless, the luminescence properties of doped Y_2_O_2_S can be improved. While most of the effort tends to be invested in optimizing the choice of dopants and their concentrations [16], the host material Y_2_O_2_S has not received much attention, and there have been only a few studies dealing with the optical properties of the pure bulk Y_2_O_2_S. Yttrium oxysulfide can be prepared as phosphor nanoparticles with infrared-to-green and blue upconversion emission [17], doped yttrium oxysulfide nanostructures [18], or quantum structures [19]. We note that an important feature of rare-earth oxysulfides is that those materials are wide-gap (4.6–4.8 eV) semiconductors [20]. The calculations for crystalline Y_2_O_2_S show that the material has an indirect bandgap and that the top of the valence bands exhibits an anisotropic character, i.e., an anisotropic mass of the hole in reciprocal space [20].

A promising alternative route to improve the properties of Y_2_O_2_S is to employ alternative polymorphs of yttrium oxysulfide, since such new modifications may exhibit improved electronic properties for their application as a host material in luminescence applications. To identify the potential candidates for new modifications of Y_2_O_2_S, we performed crystal structure prediction via global exploration of the energy landscape of yttrium oxysulfide, complemented by data-mining-based searches of the Inorganic Crystal Structure Database (ICSD). For the most promising structural candidates, we then investigated their electronic properties, demonstrating the richness of Y_2_O_2_S with regard to its structural and electronic features.

## 2. Computational Methods

### 2.1. Details of the Generation of Structural Candidates of Y_2_O_2_S

In order to predict new crystal structures, global optimizations on the energy landscape of Y_2_O_2_S have been performed using a fast-computable empirical two-body potential:

(1) $$V(\overset{\to }{r_{i}},\overset{\to }{r_{j}})=V(|\overset{\to }{r_{i}}-\overset{\to }{r_{j}}|)=V(r_{ij})$$

To describe the potential energy of the system,

(2) $$E_{pot}=\sum_{\langle i,j\rangle }\;V(\overset{\to }{r_{i}},\overset{\to }{r_{j}}),$$

the two-body potential consists of a van der Waals term and a damped Coulomb term:

(3) $$V(r_{ij})=\varepsilon_{vdW}\left[{\left(\frac{\sigma_{ij}}{r_{ij}}\right)}^{12}-{\left(\frac{\sigma_{ij}}{r_{ij}}\right)}^{6}\right]+e^{-\mu r_{ij}}\frac{q_{i}q_{j}}{4\pi \varepsilon_{0}r_{ij}}$$

with μ = 1/0.1 Å and ε_vdW_ = 0.5 eV. Here,

(4) $$\sigma_{ij}=r_{ion}(i)+r_{ion}(j)$$

is the sum of the effective ionic radii of the ions i and j, and q_i_ is the charge assigned to ion i.

In this study, we employ q(Y) = +3, q(O) = −2 and q(S) = −2 for the charges. The starting source of the radius parameters for the ions are crystallographic effective ionic radii, in particular those listed in the book by Emsley [21], with r(Y^3+^) = 1.06 Å, r(O^2−^) = 1.32 Å and r(S^2−^) = 1.94 Å. For the purpose of their use in the empirical potential, the radii are multiplied by a scale factor, r_s, that is close to one, where the values of r_s are based on experience, and for special situations a range of r_s values are employed [22]. In this study, we use r_s = 1.1 for the Y and O ions, yielding r_ion_(Y) = 1.166 Å and r_ion_(O) = 1.452 Å for the effective radii of Y and O in the expression for the empirical potential. For the sulfur ions, we use two different radius parameters in the potential, r_s = 1.1 and r_s = 1.0, i.e., r_ion_(S) = 2.134 Å and r_ion_(S) = 1.94 Å, during the global searches on the energy landscape, i.e., we perform half of the simulated annealing runs with r_ion_(S) = 2.134 Å and the other half with r_ion_(S) = 1.94 Å. The reason is that the sulfur ion exhibits a rather large range of effective ionic radii in sulfide compounds, which should be reflected in searches for structural candidates; such a parameter variation has proven quite effective in determining structural candidates in ionic-type systems [22]. The infinite crystal structure is approximated with a periodic approximant consisting of Z = 2 formula units of Y_2_O_2_S inside a periodically repeated variable unit cell. Stochastic simulated annealing was employed as the global optimization technique, where both the positions of the atoms inside the unit cell and the unit cell parameters were allowed to be varied at random during every run. During each simulated annealing run, multiple local optimizations (stochastic quenches) were performed, yielding a total of 5000 structural candidates.

The global searches were complemented by data-mining-based searches of the Inorganic Crystal Structure Database (ICSD, version 5.6.0, Data Release 2026.1) [23] to identify additional structural candidates with the general formula AB_2_X_2_, following a procedure that has been successfully applied in the Ce_2_ON_2_ chemical system [24]. The data-mining process followed the structured Knowledge Discovery in Databases (KDD) framework. This process was executed through four sequential phases: (1) selection of the primary dataset, (2) preprocessing, (3) transformation, and (4) evaluation to interpret the final patterns [24]. The structural candidates obtained via the global optimization and data mining were analyzed for their symmetries and structural similarities using the SFND [25], RGS [26], and CMPZ [27] algorithms implemented in the KPLOT software (v.9.6.15) [28], while the VESTA software (Version 3) [29] was employed to visualize the crystal structures. In particular, duplicate candidates were eliminated using the CMPZ algorithm. Promising candidates were identified based on their energy, the frequency of their appearance during global landscape explorations, and the degree of symmetry in their structures.

Afterwards, a local optimization was performed for all promising candidates (around one hundred structural candidates from global optimization and data mining searches) using ab initio calculations. This was followed by another round of structural analysis, which generated the final set of the most relevant feasible new modifications in the Y_2_O_2_S system.

### 2.2. Details of the Ab Initio Calculations

All total-energy and electronic-structure calculations were performed at the ab initio level using density functional theory (DFT) with the CRYSTAL code (versions 17 and 23) [30,31]. The local optimizations employed analytical gradients with respect to the atom positions [32] and cell parameters [33] and used a local optimization routine [34]. As a result, atoms can freely move without symmetry constraints, and cell parameters, especially cell sizes, are fully relaxed, and the cells can change shape and size [35]. All-electron basis sets (AEBSs) based on Gaussian-type orbitals (GTOs) were applied. In the case of yttrium, a [*7s5p3d*] basis set (Y_buljan_1999) was used as in refs. [36,37]. For *S*^2−^, a [*5s4p1d*] basis set was employed as in refs. [38,39], while for *O*^2−^, a [*4s3p*] basis set was applied as in refs. [40,41]. We tested additional Effective Core Potentials (ECPs) to include relativistic effects; however, they had no significant influence. Spin-Orbit Coupling (SOC) was not included in the calculations. Tolerances for the convergence of total energy are set to 10^−7^ E_h_ in structural and electronic property calculations. Furthermore, a *k*-point sampling net of size 8 × 8 × 8 with the Monkhorst–Pack scheme was employed for each modification [42]. We used a 30% Fock/KS mixing parameter (damping factor, α = 0.30) to stabilize Self-Consistent Field (SCF) convergence.

In particular, several different functionals have been employed for the DFT calculations: the Local-Density Approximation (LDA-PZ), with a parametrization as in [43], and the Generalized Gradient Approximation (GGA-PBE), with a parametrization as in [44], and the hybrid B3LYP (Becke, three-parameter, Lee–Yang–Parr) functional [45]. We note that repeating the calculations with several ab initio computational methods for total-energy calculations, local minimizations, and electronic-structure calculations facilitates a robust assessment of data accuracy and the viability of structural candidates [46,47,48].

By performing a series of isotropic structural deformations, we have computed the total ground-state energy (*E*) as a function of the cell volume (*V*). The *E*(*V*) curves serve as a critical tool for mapping the energy landscapes of inorganic crystals and predicting phase stability under pressure. We used the CRYSTAL program to perform an automated volume scan to compute *E* vs. *V* curves, which we then fit to various universal Equations of State (EOS). For each volume, we performed a full *V*-constrained geometry optimization. Finally, we used a third-order isothermal Birch–Murnaghan fit and a 4th-order polynomial fitting procedure to compute equilibrium properties and *E*(*V*) and *H*(*p*) curves.

## 3. Results

### 3.1. Energy Landscape and Crystal Structures

Overall, 5000+ structural candidates were generated by the global landscape search and the data-mining procedure. After relaxing the most promising among these candidates on an ab initio (DFT) level, the four structures with the lowest energy belonged to the results of the global optimization, and one further low-energy candidate (*ε*-Y_2_O_2_S) to the outcome of the data-mining-based searches.

The stability of the predicted crystal structures of Y_2_O_2_S is assessed using their computed total energies at the ab initio level. The modification with the lowest energy is the thermodynamically most stable one at standard conditions, i.e., for essentially zero pressure and low temperatures, while the higher-lying local minima of the energy serve as candidates for metastable phases at standard conditions and/or as possible candidates for high-temperature, high-pressure, or effective negative-pressure phases. A further investigation of stability and metastability is possible using phonon calculations or other dynamical-stability calculations.

**Figure 1:**
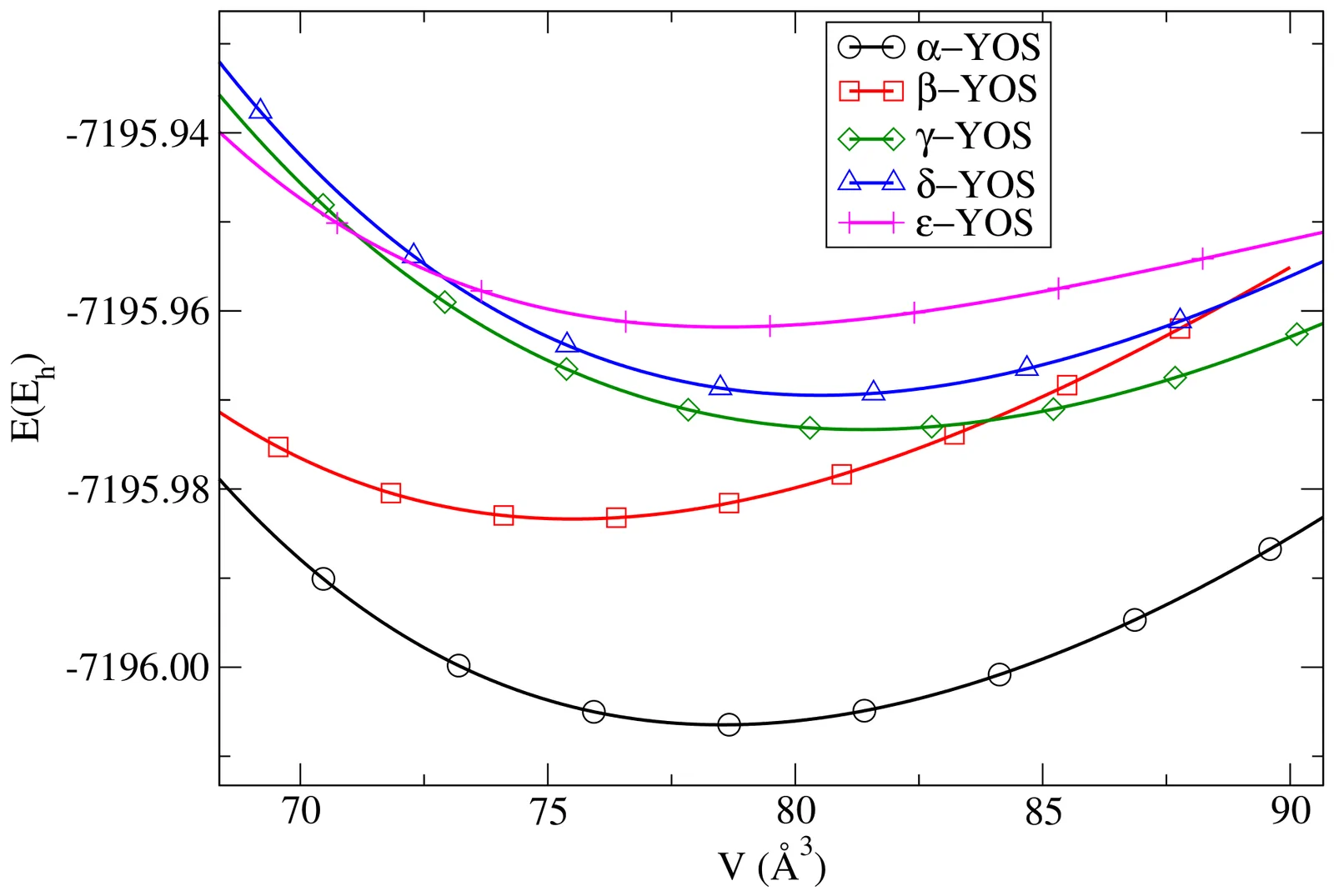
Computed *E*(*V*) curves for the five most relevant predicted crystal structures in the Y_2_O_2_S system. The calculations were performed using the LDA-PZ functional.

In Figure 1, we show the *E*(*V*) curves for the five most relevant predicted structures of Y_2_O_2_S, i.e., the ones exhibiting the lowest energies, computed using the LDA-PZ method. We note that the *α*-phase (*α*-Y_2_O_2_S) corresponds to the lowest energy minimum (global minimum) and thus represents the thermodynamically most stable structure, in agreement with the data in the literature [2,8,20,49,50]. Next in energy ranking is the predicted *β*-Y_2_O_2_S polymorph, followed by the *γ*-Y_2_O_2_S and *δ*-Y_2_O_2_S modifications, while the *ε*-Y_2_O_2_S modification appears as the highest energy minimum regardless of computational approach (Table 1). A summary of the computed total energy per formula unit of these five structures, where the ab initio calculations were performed using the LDA-PZ, B3LYP, and GGA-PBE approximations, is shown in Table 1 (relative energies are given in the Supporting Information Table S1). We note that the ranking of the minima by energy does not change when we employ a different functional, indicating the robustness of the results.

**Figure 2:**
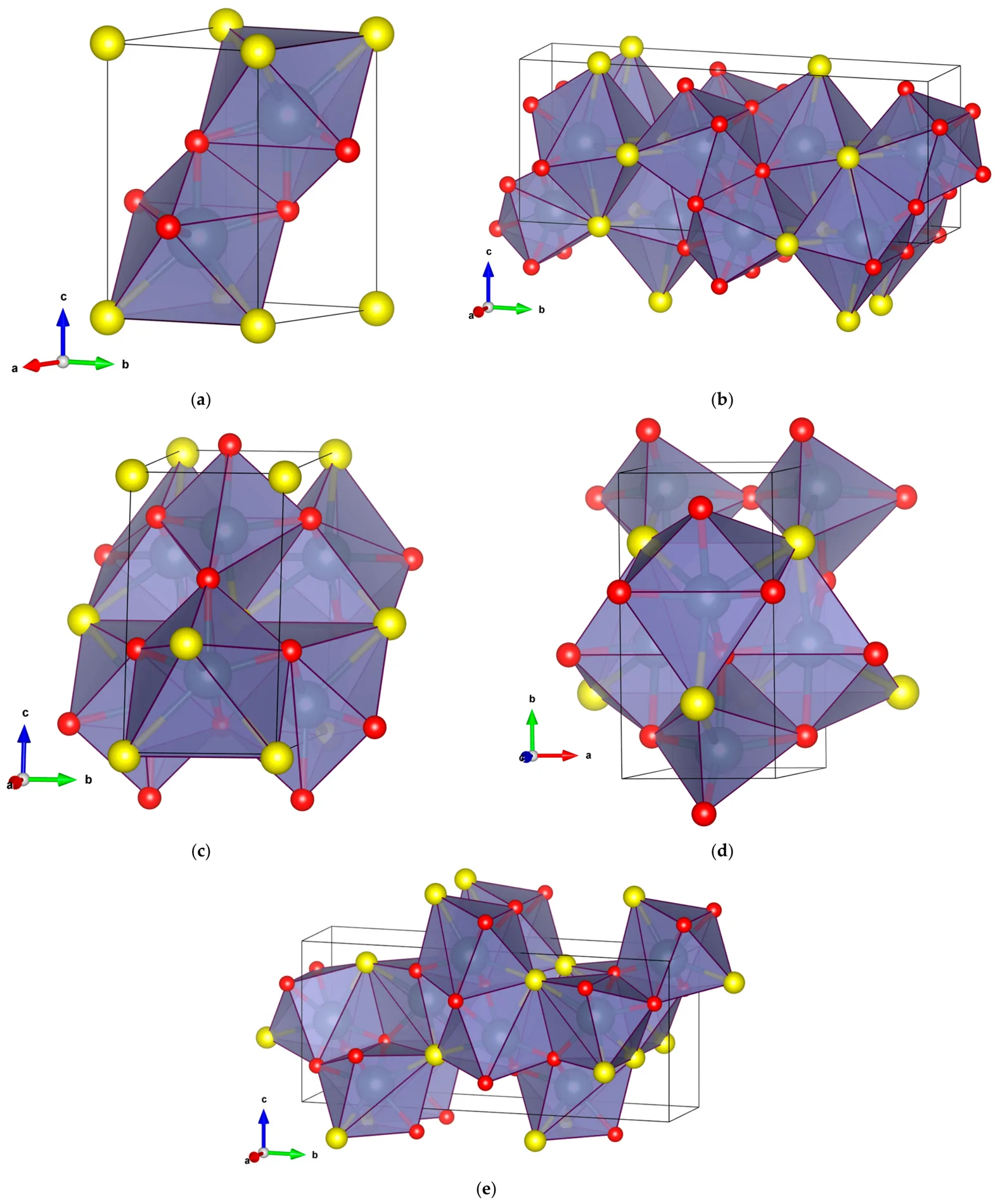
Crystal structure of the five most promising candidate modifications of Y_2_O_2_S: (**a**) *α*-Y_2_O_2_S; (**b**) *β*-Y_2_O_2_S; (**c**) *γ*-Y_2_O_2_S; (**d**) *δ*-Y_2_O_2_S; (**e**) *ε*-Y_2_O_2_S. Yttrium, sulfur, and oxygen atoms are denoted by violet, yellow, and red spheres, respectively.

The *α*-phase of yttrium oxysulfide obtained in this study as the global minimum on the energy landscape corresponds to the experimentally known phase of Y_2_O_2_S [2,8,20,49,50] and is a global minimum on the energy landscape (Figure 1 and Table 1). The crystal symmetry of Y_2_O_2_S is trigonal, with the space group *P*-3*m*1 (no. 164). The *α*-Y_2_O_2_S structure consists of edge-connected seven-fold coordination polyhedra around the yttrium atom, which is surrounded by four oxygen atoms with an average distance of 2.23 Å and three sulfur atoms at a distance of 2.83 Å (Figure 2a). The computed lattice constants are *a* = 3.83 Å and *c* = 6.69 Å using B3LYP; a = 3.80 Å and c = 6.67 Å using GGA-PBE; and a = 3.73 Å and c = 6.54 Å using LDA-PZ (Table 2).

The predicted *β*-Y_2_O_2_S modification is a metastable phase of yttrium oxysulfide that appears in orthorhombic symmetry with the *Cmc*2_1_ (no. 36) space group. This structure was frequently found in the global searches using empirical potentials; the ab initio optimization resulted in the lattice constants *a* = 3.65 Å, *b* = 15.22 Å, *c* = 5.93 Å using B3LYP; *a* = 3.64 Å, *b* = 15.14 Å, *c* = 5.88 Å using GGA-PBE; and *a* = 3.56 Å, *b* = 14.96 Å, *c* = 5.70 Å using LDA-PZ (Table 2). The structure of *β*-Y_2_O_2_S includes two types of edge-connected coordination polyhedra around the yttrium atoms. The first type is a seven-fold coordination polyhedron, with three oxygen atoms and four sulfur atoms (Figure 2b). The average bond distance is 2.57 Å. The second type is an 8-fold coordination polyhedron, where yttrium is surrounded by six oxygen and two sulfur atoms, and the average cation–anion distance is 2.49 Å (Figure 2b).

The structure of the *γ*-Y_2_O_2_S phase exhibits the lowest symmetry among the five structures with the lowest energies, appearing in the monoclinic *Pm* (no. 6) space group. In γ-Y_2_O_2_S, yttrium is surrounded by edge-connected five- and seven-fold coordination polyhedra (Figure 2c). In the five-fold coordination polyhedra, the average bond distance is 2.36 Å, while in the seven-fold coordination polyhedra, it varies between 2.49 and 2.54 Å. The unit cell parameters of the *γ*-phase of yttrium oxysulfide are a = 6.19 Å, b = 3.99 Å, c = 7.26 Å, β = 100.64° using B3LYP; a = 6.17 Å, b = 3.97 Å, c = 7.21 Å, β = 100.46° using GGA; and a = 6.05 Å, b = 3.89 Å, c = 7.02 Å, β = 99.94° using LDA (Table 2).

The predicted δ-phase of yttrium oxysulfide also shows an orthorhombic symmetry similar to the *β*-phase, but with a different space group, i.e., the δ-Y_2_O_2_S modification appears in the *Pmn*2_1_ (no. 31) space group. The calculated unit cell parameters are *a* = 3.82 Å, *b* = 7.50 Å, *c* = 6.09 Å using B3LYP; *a* = 3.80 Å, *b* = 7.43 Å, *c* = 6.08 Å using GGA-PBE; and *a* = 3.71 Å, *b* = 7.25 Å, *c* = 5.98 Å using LDA-PZ (Table 2). The *δ*-Y_2_O_2_S structure is based on edge-connected five- and seven-fold coordination polyhedra around the yttrium atoms, similar to the *γ*-Y_2_O_2_S modification (Figure 2d). In the five-fold coordination polyhedra around the yttrium atoms, the average anion–cation distance is 2.36 Å, while for the seven-fold coordination polyhedra it is 2.48 Å.

Finally, the ε-Y_2_O_2_S modification exhibits an orthorhombic symmetry with the same space group as the *β*-phase, i.e., *Cmc*2_1_ (no. 36). However, the ε-phase is structurally distinct from the *β*-phase and all previous ones: the *ε*-Y_2_O_2_S structure consists of two types of edge-connected seven-fold coordination polyhedra around the yttrium atoms. The first type of these polyhedra shows an average cation–anion bond distance of 2.47 Å (four oxygen and three sulfur atoms), and in the second type (five oxygen and two sulfur atoms), the average bond distance is 2.50 Å (Figure 2e). A summary of all the predicted crystal structures of Y_2_O_2_S computed using the B3LYP, GGA-PBE, and LDA-PZ functionals is shown in Table 2.

### 3.2. Electronic Band-Structure Calculations

To explore the potential of the Y_2_O_2_S system for applications, the electronic properties and the size of the bandgap of the predicted theoretical modifications of Y_2_O_2_S were investigated and compared with the modifications reported in the literature. The DFT calculations were performed using the GGA-PBE, the LDA-PZ, and the B3LYP hybrid functionals, as in the structural relaxations.

**Figure 3:**
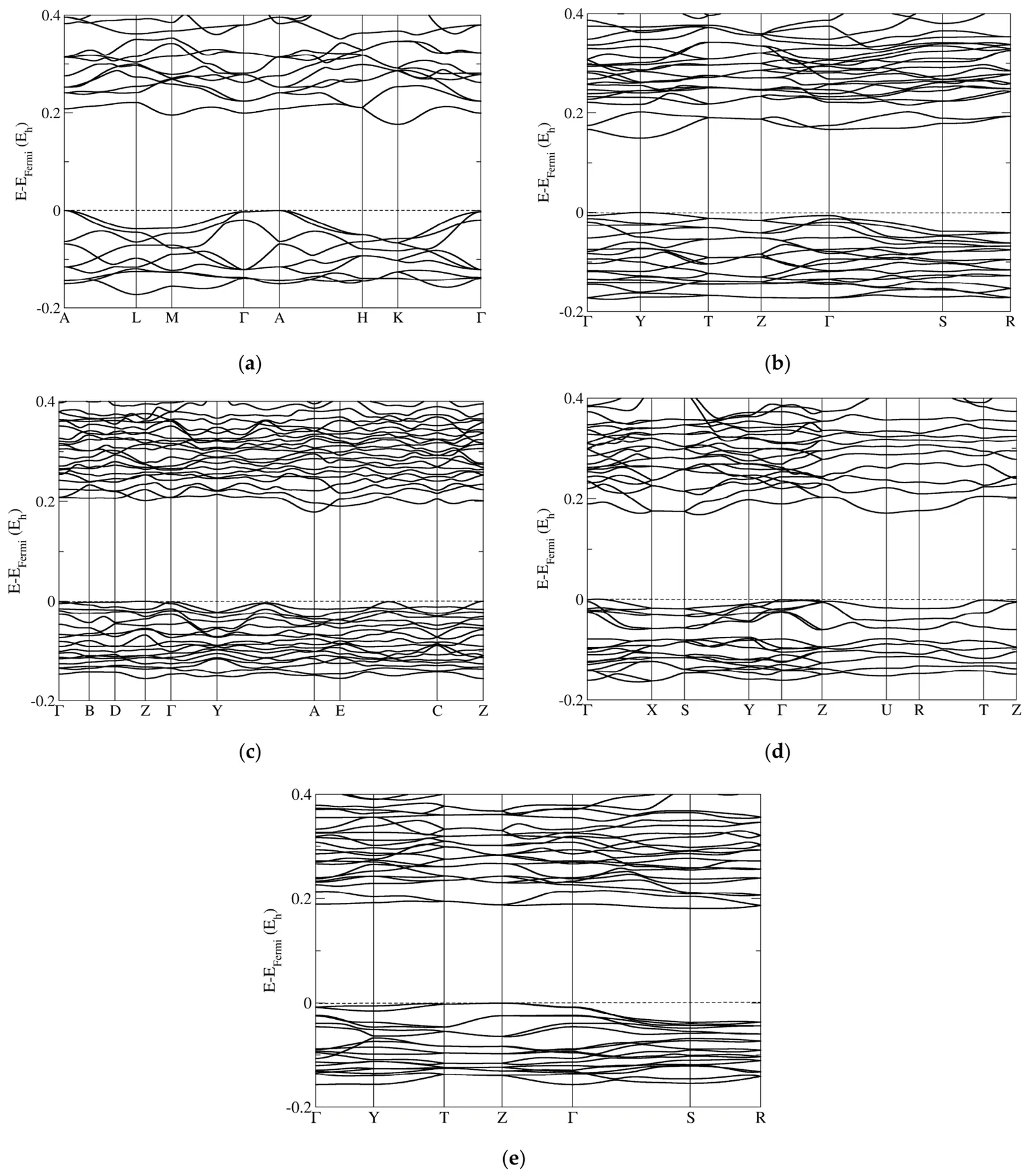
Band structure of the five most promising Y_2_O_2_S modifications calculated with the hybrid B3LYP functional: (**a**) *α*-Y_2_O_2_S; (**b**) *β*-Y_2_O_2_S; (**c**) *γ*-Y_2_O_2_S; (**d**) *δ*-Y_2_O_2_S; (**e**) *ε*-Y_2_O_2_S. The analogous band structures computed using the LDA-PZ functional are shown in Figure S1 in the Supplementary Materials.

Figure 3a shows the electronic band structures of the trigonal *α*-Y_2_O_2_S crystallizing in the *P-3m1* (no. 164) space group, calculated along the high-symmetry path A-L-M-Γ–A-H–K–Γ of the Brillouin zone, as defined in ref. [51]. In the case of the α-modification, the valence band maximum (VBM) is located at the A point of the Brillouin zone, while the conduction band minimum (CBM) is located at the K point, indicating an indirect bandgap of 4.92 eV computed using the hybrid B3LYP functional (Figure 3a), and 3.23 eV on the LDA-PZ level of calculation (Figure S1a). This is in good agreement with the literature data [20,52,53,54,55,56,57].

Next, Figure 3b shows the band structure of the predicted *β*-phase of yttrium oxysulfide. The *β*-Y_2_O_2_S structure appears in the orthorhombic *Cmc*2_1_ (no. 36) space group, and we have computed the band structure along the high-symmetry path Γ–Y-T-Z-Γ-S-R of the Brillouin zone. In contrast to the *α*-phase, the VBM and CBM are both located at the Y point of the Brillouin zone in the *β*-modification, showing a direct bandgap of 4.07 eV using B3LYP and 2.28 eV using the LDA-PZ functional (Figure 3b and Figure S1b). We note that a possible indirect-to-direct bandgap transition associated with the switch between these two (meta)stable phases of Y_2_O_2_S could be of great importance in optoelectronic applications.

The electronic band structure of the low-symmetry monoclinic phase of yttrium oxysulfide (*γ*-Y_2_O_2_S) crystallizing in space group *Pm* (no. 6) is discussed next (Figure 3c). The calculations were performed along the path Γ–B-D-Z–Γ-Y-A-E-C-Z of the Brillouin zone. This monoclinic structure exhibits an indirect bandgap, where the VBM is located at the Γ point, and the CBM is located at the A point of the Brillouin zone with a bandgap size of 4.86 eV, calculated using the hybrid B3LYP method. Moreover, the indirect bandgap character was confirmed in the calculations using the LDA-PZ functional, yielding a bandgap of 3.17 eV (Figure S1c).

Figure 3d shows the results of the band-structure calculations of the orthorhombic *δ*-Y_2_O_2_S phase using the B3LYP functional. Since the *δ*-Y_2_O_2_S structure appears in space group *Pmn2*_1_ (no. 31), the band structure is calculated along the complicated high-symmetry path Γ–X–S–Y–Γ-Z-U-R-T-Z of the Brillouin zone; this path is different from the one used for the *β*-phase, which also crystallizes in an orthorhombic structure but in a different space group. The *δ*-Y_2_O_2_S phase shows an indirect bandgap, where the VBM is located at the Γ point, and the CBM is located along the Z-U directions of the Brillouin zone. The indirect bandgap was found to be 4.58 eV using the hybrid B3LYP functional. An indirect bandgap of 2.80 eV was also found using the LDA-PZ approximation (Figure S1d).

Finally, the electronic band structure of the *ε*-Y_2_O_2_S modification is shown in Figure 3e. The *ε*-Y_2_O_2_S structure appears in the orthorhombic *Cmc*2_1_ (no. 36) space group; since this is the same as that of the *β*-Y_2_O_2_S modification, we have computed the band structure along the same high-symmetry path Γ–Y-T-Z-Γ-S-R of the Brillouin zone (cf. Figure 3b,e). In the case of the *ε*-phase, the VBM is located at the Z point, and the CBM is located at the Y point of the Brillouin zone, with a bandgap size of 4.91 eV using the B3LYP and 2.35 eV using the LDA-PZ functional (Figure 3e and Figure S1e and Table 3). We note that within the same chemical system (Y_2_O_2_S), we deal with two different modifications that exhibit the same symmetry and space group, and thus they might be expected to exhibit a similar qualitative band structure; however, the *β*-Y_2_O_2_S phase has a direct bandgap, while the ε-Y_2_O_2_S modification has an indirect bandgap. Thus, creating intergrowth structures that partake in both of these modifications might open new avenues for optoelectronic applications, e.g., possibly allowing controlled switching between indirect and direct bandgap behavior in a material.

The calculated bandgaps obtained using different exchange–correlation functionals are summarized in Table 3 and compared with previously reported theoretical and experimental values, where available.

## 4. Discussion

The α-modification, the only experimentally known phase of yttrium oxysulfide (Y_2_O_2_S), has received considerable attention as a phosphorescent host material and is widely used in the luminescent materials industry [2,49,50,54,55]. However, both with regard to the lattice constants and the bandgap values, not many experimental or theoretical data are available, and some disagreement exists among them. This is because isolating Y_2_O_2_S requires careful control of synthesis conditions, sulfurization potential, reaction templates, and thermal kinetics. The lattice constants computed in this study are a = 3.83 Å and c = 6.69 Å using the B3LYP functional, a = 3.80 Å and c = 6.67 Å using the GGA-PBE functional, and a = 3.73 Å and c = 6.54 Å using the LDA-PZ functional (Table 2). The previous theoretical data show a = 3.750 Å, c = 6.525 Å [2], where the exchange–correlation energy is evaluated within the local density approximation (LDA) by using Ceperley–Alder homogeneous electron gas data [58], and a = 3.730 Å, c = 6.48 Å [49], by using the hybrid density functional sX-LDA for electronic structure calculations under the framework of the generalized Kohn–Sham density functional theory (GKS-DFT) [59]. Those calculated literature data are in close agreement with our LDA-PZ calculations. In contrast, the experimental reports of a = 3.7826 Å, c = 6.5689 Å [54,55] and a = 3.784 Å, c = 6.586 Å [50] agree better with our present hybrid B3LYP and GGA-PBE results, consistent with the usually larger lattice parameters at the B3LYP and GGA level, compared to LDA-PZ calculations (cf. Table 2).

In this context, we note that one usually obtains slightly larger lattice parameters at the GGA level than in LDA-PZ calculations. On the other hand, LDA- and GGA-level bandgap calculations frequently underestimate the true bandgap; for these systems, the value obtained with B3LYP is often preferable. In particular, we further note that the location of the valence bands obtained from the DFT and especially from the GGA-PBE calculations is usually accurate, while the energy levels of the conduction bands are sometimes systematically underestimated, an aspect that needs to be kept in mind when comparing experimental bandgaps with computed ones using certain functionals. As a consequence, some studies explicitly adjust the computed bandgap by adding or subtracting some constant on the order of 1–2 eV; however, applying such a shift as a standard modus operandi strikes us as not really justified.

As far as the electronic properties are concerned, the *α*-phase of Y_2_O_2_S is a WBG (4.6–4.8 eV) semiconductor that has been considered an ideal host matrix for trivalent rare-earth cations [4,52], as mentioned in the Introduction. However, Y_2_O_2_S is a specific wide-bandgap semiconductor that differs from more industrially accepted and advanced WBG semiconductors such as AlN [60,61,62]. Moreover, an X-ray photoelectron spectroscopy study was conducted to obtain more detailed information on the valence band and the outermost core band, and the bandgap energy was estimated at 6.77 eV [53], which greatly exceeds the commonly accepted value. On the other hand, Li et al. believe that the bandgap energy of 6.77 eV reported by Itoh and Inabe is overestimated and that the true bandgap should be around 4.6 eV (Table 3) [57]. Furthermore, in previous theoretical calculations, the bandgap of Y_2_O_2_S was estimated at about 3.0 eV, which is 1.6 eV lower than the commonly accepted experimental value [8]. Assuming that the valence bands obtained from their DFT calculations were accurate and that the conduction bands were underestimated, a “scissor operation” of 1.6 eV was adopted in the conduction band so that the calculated bandgap agreed with the experimental value [63].

In the present study, the computed bandgap values were 3.23 eV (LDA), 3.46 eV (GGA), and 4.92 eV (B3LYP) for the α-Y_2_O_2_S modification. A survey of the existing literature underscores the pronounced impact of the exchange–correlation functional on bandgap calculations. Furthermore, considering the data for all five modifications in Table 3 for the three functionals employed demonstrates that the bandgap estimates for a given structural modification vary substantially across the LDA, GGA, and hybrid-functional approaches. In particular, the present GGA and LDA calculations agree very well with the previous theoretical data for the α-modification, while the hybrid B3LYP calculations agree with the experimental data (Table 3). We also note that for each of the five modifications considered in this study, the smallest bandgap is obtained with LDA-PZ, while the largest bandgap is obtained with the hybrid B3LYP functional. However, the ranking of the size of the four indirect bandgaps was not the same for the three functionals: in the case of B3LYP, the order was E_gap_(α) > E_gap_(ε) > E_gap_(γ) > E_gap_(δ). On the other hand, for GGA-PBE, we find E_gap_(ε) > E_gap_(α) > E_gap_(γ) > E_gap_(δ), and for LDA-PZ we have E_gap_(α) > E_gap_(γ) > E_gap_(δ) > E_gap_(ε). This clearly demonstrates the remaining uncertainty implicit in the functionals typically used for bandgap calculations; nevertheless, the general trend is clear, and the differences in the energy gaps that lead to a different ranking are small. To further analyze bandgaps and perform comparative assessments of direct/indirect bandgap transitions, one can turn to Spin-Orbit Coupling (SOC) methodology [64,65], the Tran–Blaha modified Becke–Johnson (TB-mBJ) functional [66], or Time-Dependent Density Functional Theory (TD-DFT) calculations [67].

However, regardless of the quantitative details of the computed bandgaps of the various feasible modifications we have considered in this study, the results clearly show that a tuning of about 1 eV in the bandgap should be possible in the yttrium oxysulfide system by switching from one (meta)stable phase to another. Beyond the fine-tuning of the indirect bandgap by using the α, γ, δ and ε modifications, the discovery of a direct-gap polymorph in the Y_2_O_2_S system, the β-phase, is of great importance and opens new possibilities for applications of Y_2_O_2_S beyond its traditional use as a phosphor host. The present results suggest that phase control could serve as a viable route for engineering the electronic properties of Y_2_O_2_S. In particular, the β-phase, with its direct bandgap, may be of interest for applications in thin-film transistors, light-emitting diodes, or photovoltaic devices, provided that synthesis routes can be developed to stabilize this metastable modification.

**Figure 4:**
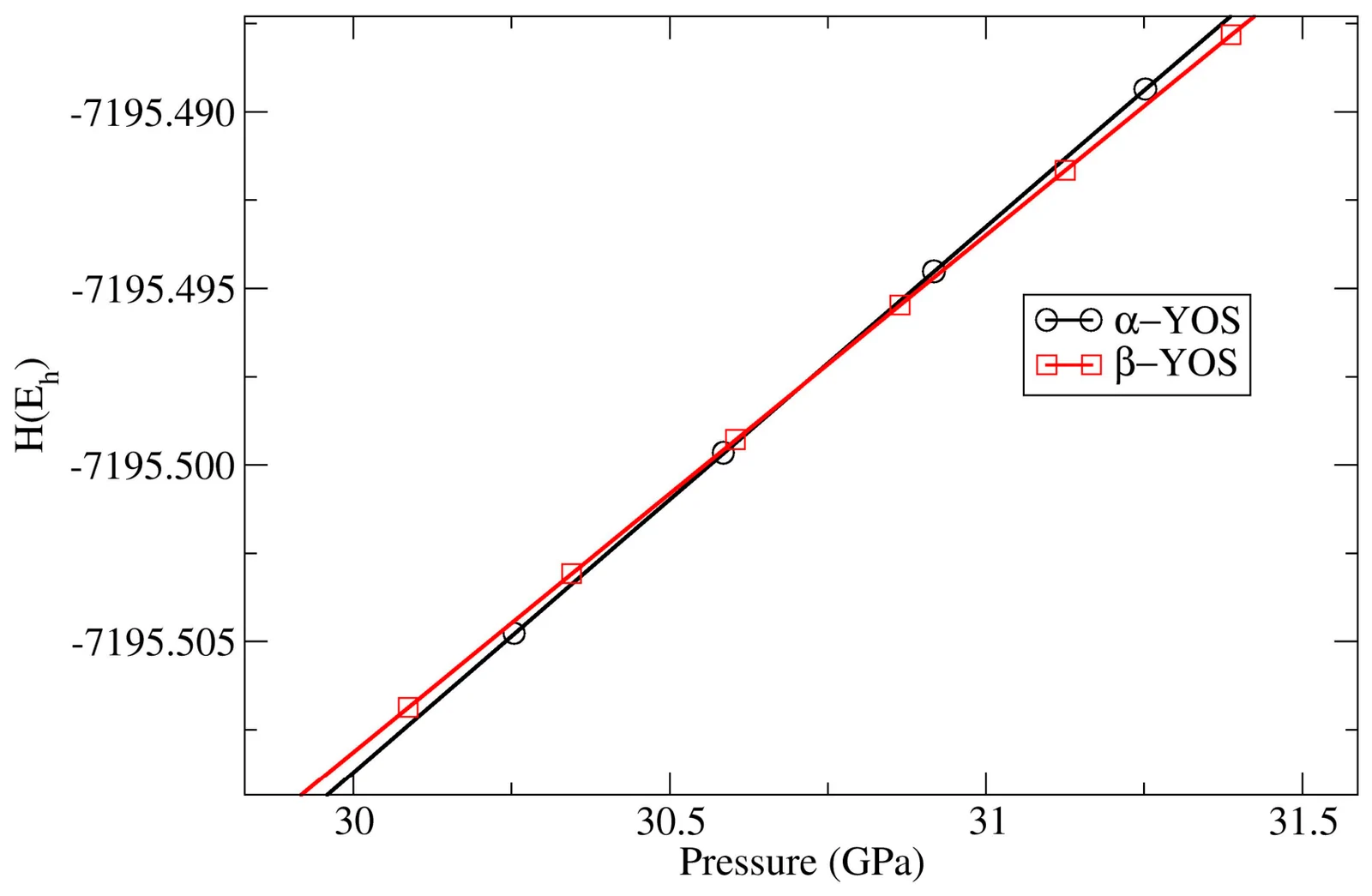
Computed *H*(*p*) curves for the α- and β-type crystal structures in the Y_2_O_2_S system. The calculations were performed using the LDA-PZ functional.

While this clearly poses a major challenge to the experimental solid-state chemist and the materials scientist, the *E*(*V*) curves in Figure 1 already indicate that the β-Y_2_O_2_S modifications should be the high-pressure phase in the Y_2_O_2_S system. In addition, we have computed the enthalpy–pressure (*H*(*p*)) curves and determined the α → β transition pressure of approximately 30.8 GPa using the LDA-PZ functional (Figure 4), and around 35 GPa using the GGA-PBE and 40 GPa using the B3LYP functional. Other possible synthesis routes might be designed following the recent combined chemistry approach, in which structure–prediction calculations and advanced electron microscopy techniques demonstrate the topochemical deintercalation/reintercalation of sulfur in a layered oxychalcogenide, suggesting additional options for the future design of novel metastable phases [68].

Synthesizing the *β*-phase would allow the realization of the indirect-to-direct bandgap transition (i.e., the *α* → *β* phase transition) we predict as being feasible in the Y_2_O_2_S system, which is of great importance in optoelectronic applications. This type of tuning of electronic properties constitutes another example of a polymorph-dependent bandgap transition behavior, similar to the one that has recently been observed in La_2_O_2_S, where a newly synthesized direct-gap phase, an indirect-gap high-pressure phase, and a pressure-driven phase transition were identified [69]. Adding another example in the form of the *α* → *β* phase transition in Y_2_O_2_S strongly suggests that polymorphism-driven electronic tuning may be a general feature of rare-earth oxysulfides.

## 5. Conclusions

This study investigates the feasible crystalline phases of yttrium oxysulfide (Y_2_O_2_S) and their electronic properties using advanced computational methods. By employing crystal structure prediction, we explored the compound’s energy landscape through global optimization on the empirical potential energy landscape supplemented with data mining, followed by an ab initio refinement using the LDA-PZ, the GGA-PBE, and the B3LYP hybrid functionals.

Our results confirm the thermodynamic stability of the experimentally known α-phase, yielding a structure in excellent agreement with the experimentally observed trigonal modification. Beyond the known phase, we identified four novel metastable polymorphs (*β*-, *γ*-, *δ*-, and *ε*-Y_2_O_2_S). The electronic structure analysis characterizes the α-phase as a wide-bandgap semiconductor with an indirect bandgap, in agreement with the experimental results. Crucially, the analysis of the electronic properties of the newly predicted polymorphs shows that a high-pressure phase, the *β*-modification, exhibits a direct bandgap and has the potential to realize an indirect-to-direct bandgap transition in the Y_2_O_2_S system, highlighting the material’s versatility for optoelectronic and photovoltaic applications.

## Figures and Tables

**Table 1:** Total energy in hartrees (E_h_) per formula unit of the energetically most favorable structures at calculated equilibrium volumes. The local optimizations were performed within the LDA-PZ, the B3LYP, and the GGA-PBE approximations.

Structure Type	Space Group	B3LYP	GGA-PBE	LDA-PZ
*α*-Y_2_O_2_S	*P*-3*m*1 (no. 164)	−7205.8345	−7205.1438	−7196.0013
*β*-Y_2_O_2_S	*Cmc*2_1_ (no. 36)	−7205.8091	−7205.1228	−7195.9813
*γ*-Y_2_O_2_S	*Pm* (no. 6)	−7205.8075	−7205.1176	−7195.9726
*δ*-Y_2_O_2_S	*Pmn*2_1_ (no. 31)	−7205.7993	−7205.1132	−7195.9699
*ε*-Y_2_O_2_S	*Cmc*2_1_ (no. 36)	−7205.7937	−7205.1045	−7195.9608

**Table 2:** Computed structural data (space group and unit cell parameters) for the five most relevant crystal structures in Y_2_O_2_S. The calculations were performed using the GGA-PBE, LDA-PZ, and hybrid B3LYP functionals.

Structure Type and Space Group	Cell Parameters (Å) and Fractional Coordinates		
	B3LYP	GGA-PBE	LDA-PZ
*α*-Y_2_O_2_S *P*-3*m*1 (no. 164)	*a* = 3.83, *c* = 6.69 Y 1/3 2/3 0.7170 O 1/3 2/3 0.3722 S 0 0 0	*a* = 3.80, *c* = 6.67 Y 1/3 2/3 0.7181 O 1/3 2/3 0.3717 S 0 0 0	*a* = 3.73, *c* = 6.54 Y 1/3 2/3 0.7190 O 1/3 2/3 0.3713 S 0 0 0
*β*-Y_2_O_2_S *Cmc*2_1_ (no. 36)	*a* = 3.65, *b* = 15.22, *c* = 5.93 Y1 0 0.1575 0.5105 Y20 0.5465 0.0289 O1 0 0.4062 0.8932 O2 0 0.0283 0.7137 S1 0 0.1945 0.9984	*a* = 3.64, *b* = 15.14, *c* = 5.88 Y1 0 0.1577 0.5101 Y2 0 0.5476 0.0245 O1 0 0.4062 0.8920 O2 0 0.0285 0.7176 S1 0 0.1953 0.0006	*a* = 3.56, *b* = 14.96, *c* = 5.70 Y1 0 0.1590 0.5104 Y2 0 0.5480 0.0200 O1 0 0.4061 0.8914 O2 0 0.0289 0.7213 S1 0 0.1950 0.0016
*γ*-Y_2_O_2_S *Pm* (no. 6)	*a* = 6.19, *b* = 3.99, *c* = 7.26, *β* = 100.64 Y1 0.8849 1/2 0.2707 Y2 0.6307 1/2 0.7694 Y3 0.0949 0 0.6369 Y4 0.4431 0 0.1307 O1 0.9188 1/2 0.6170 O2 0.5709 1/2 0.0668 O3 0.4452 0 0.7961 O4 0.7830 0 0.3438 S1 0.9843 0 0.9847 S2 0.3244 1/2 0.4176	*a* = 6.17, *b* = 3.97, *c* = 7.21, *β* = 100.46 Y1 0.8845 0 0.2695 Y2 0.6323 0 0.7688 Y3 0.0983 1/2 0.6367 Y4 0.4401 1/2 0.1321 O1 0.9227 0 0.6170 O2 0.5685 0 0.0664 O3 0.4467 1/2 0.7960 O4 0.7790 1/2 0.3449 S1 0.9821 1/2 0.9846 S2 0.3260 0 0.4178	*a* = 6.05, *b* = 3.89, *c* = 7.02, *β* = 99.94 Y1 0.8831 1/2 0.2704 Y2 0.6342 1/2 0.7665 Y3 0.1016 0 0.6344 Y4 0.4379 0 0.1324 O1 0.9272 1/2 0.6154 O2 0.5685 1/2 0.0656 O3 0.4473 0 0.7965 O4 0.7738 0 0.3507 S1 0.9780 0 0.9854 S2 0.3286 1/2 0.4164
*δ*-Y_2_O_2_S *Pmn*2_1_ (no. 31)	*a* = 3.82, *b* = 7.50, *c* = 6.09 Y1 1/2 0.0877 0.5972 Y2 1/2 0.5898 0.7943 O1 0 0.6109 0.0070 O2 0 0.1169 0.4090 S1 1/2 0.2526 0.0022	*a* = 3.80, *b* = 7.43, *c* = 6.08 Y1 1/2 0.0818 0.5956 Y2 1/2 0.5874 0.7954 O1 0 0.6106 0.0076 O2 0 0.1182 0.4070 S1 1/2 0.2486 0.0041	*a* = 3.71, *b* = 7.25, *c* = 5.98 Y1 1/2 0.0778 0.5940 Y2 1/2 0.5854 0.7975 O1 0 0.6112 0.0088 O2 0 0.1189 0.4032 S1 1/2 0.2459 0.0061
*ε*-Y_2_O_2_S *Cmc*2_1_ (no. 36)	*a* = 3.85, *b* = 15.19, *c* = 6.24 Y1 0 0.2015 0.1231 Y2 0 0.4407 0.9412 O1 0 0.7010 0.3340 O2 0 0.0647 0.2779 S1 0 0.3749 0.3412	*a* = 3.75, *b* = 14.96, *c* = 6.23 Y1 0 0.2007 0.1259 Y2 0 0.4468 0.9491 O1 0 0.6963 0.3415 O2 0 0.0554 0.2616 S1 0 0.3706 0.3393	*a* = 3.54, *b* = 14.50, *c* = 6.11 Y1 0 0.1995 0.1309 Y2 0 0.4578 0.9653 O1 0 0.6874 0.3542 O2 0 0.0405 0.2298 S1 0 0.3643 0.3372

**Table 3:** Summary of the computed bandgaps of the investigated yttrium oxysulfide phases using three different exchange–correlation functionals. Bandgap values are given in eV. (I) indicates an indirect bandgap.

Structure Type	Bandgap (eV)				
	B3LYP	GGA-PBE	LDA-PZ	Previous Experiment	Previous Theory
*α*-Y_2_O_2_S	4.92 (I)	3.46 (I)	3.23 (I)	4.60 ^a^; 6.77 ^b^	3.01 ^c^; 3.80 ^c^; 2.61 ^d^; 2.979 ^e^; 4.60 ^f^
*β*-Y_2_O_2_S	4.07	2.58	2.28	n.a.	n.a.
γ-Y_2_O_2_S	4.86 (I)	3.34 (I)	3.17 (I)	n.a.	n.a.
*δ*-Y_2_O_2_S	4.58 (I)	3.01 (I)	2.80 (I)	n.a.	n.a.
*ε*-Y_2_O_2_S	4.91 (I)	3.50 (I)	2.35 (I)	n.a.	n.a.

Ref. ^a^ [52]; ^b^ [53]; ^c^ [54,55]; ^d^ [20]; ^e^ [56]; ^f^ [57].

## Data Availability

The original contributions presented in this study are included in the article/Supplementary Materials. Further inquiries can be directed to the corresponding authors.

## Supplementary Materials

The following supporting information can be downloaded at: https://www.mdpi.com/article/10.3390/ma19184018/s1, Figure S1. Band structure of the five most promising Y_2_O_2_S modifications calculated with the LDA-PZ functional: (a) *α*-Y_2_O_2_S; (b) *β*-Y_2_O_2_S; (c) *γ*-Y_2_O_2_S; (d) *δ*-Y_2_O_2_S; (e) *ε*-Y_2_O_2_S. Table S1. Relative energies, ΔE with respect to α-Y_2_O_2_S, in electron volts (eV) per formula unit of the energetically most favorable structures at calculated equilibrium volumes. The local optimizations were performed within the LDA-PZ, B3LYP, and GGA-PBE approximations.
