# Supplementary material for: Energy Landscape and Electronic Properties of Yttrium Oxysulfide (Y2O2S)
Source: Materials (Basel). 2026 Sep 21;19(18):4018. doi: 10.3390/ma19184018 (PMC13609415; doi:10.3390/ma19184018)
Supplement: Supplementary file 1 [file materials-19-04018-s001.zip › materials-4561099-supplementary.pdf]

Energy Landscape and Electronic Properties of Yttrium Oxysulfide (Y_2O_2S)

Dragana Jordanov [1], Jelena Zagorac [1], Johann Christian Schön [2,*], Klaus Doll [3] and Dejan Zagorac [1,*]

[1] Materials Science Laboratory, Institute of Nuclear Sciences Vinča, University of Belgrade, 11351 Belgrade, Serbia

[2] Max Planck Institute for Solid State Research, 70569 Stuttgart, Germany

[3] Institute of Theoretical Chemistry, University of Stuttgart, 70569 Stuttgart, Germany

Correspondence: c.schoen@fkf.mpg.de (J.C.S.); dzagorac@vinca.rs (D.Z.)

Supporting Information

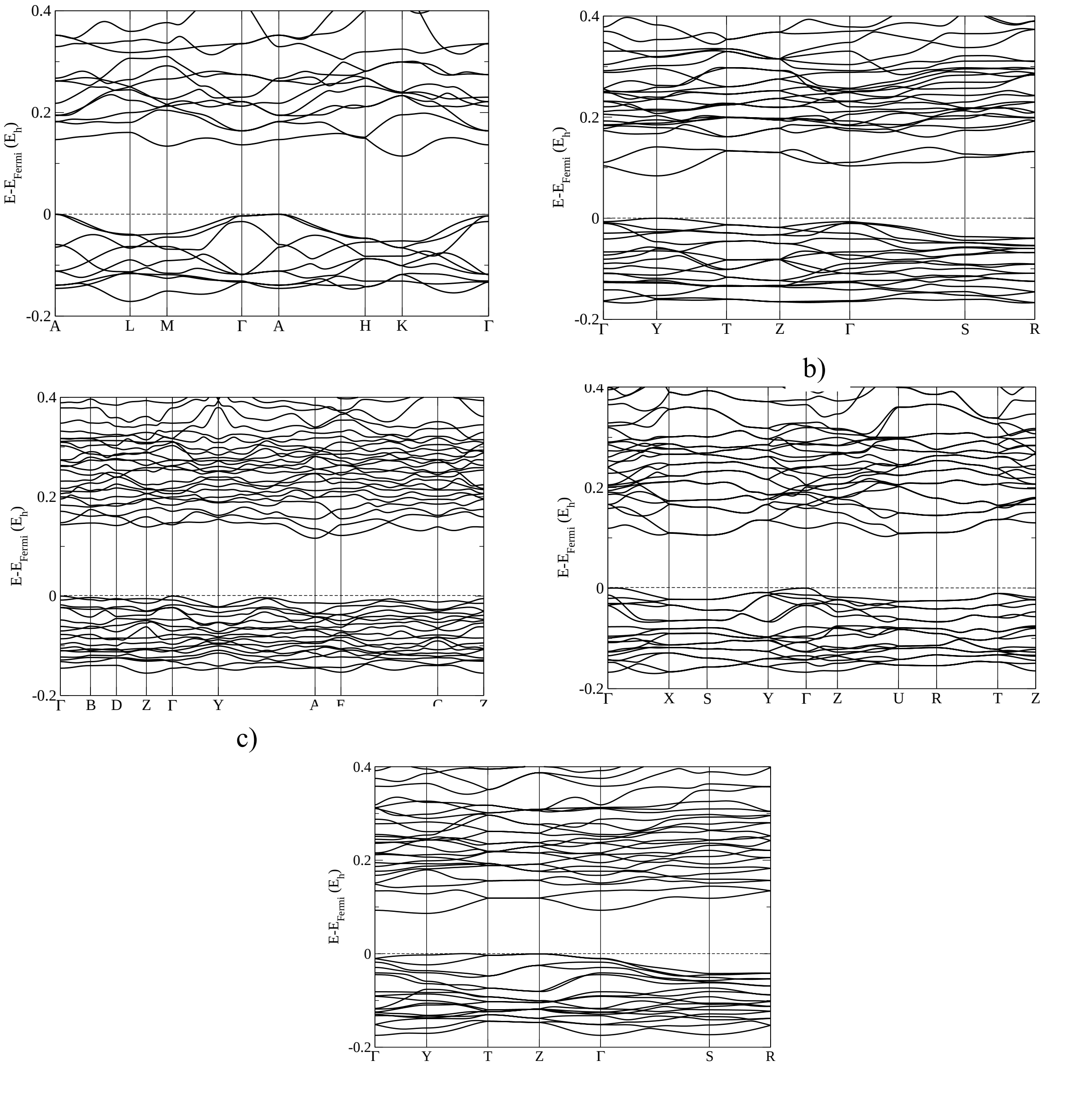

Figure S1. Band structure of the five most promising Y_2O_2S modifications calculated with the LDA-PZ functional: a) α-Y_2O_2S; b) β-Y_2O_2S; c) γ-Y_2O_2S; d) δ-Y_2O_2S; e) ε-Y_2O_2S.

Table S1. Relative energies, ΔE with respect to α-Y_2O_2S, in electron volts (eV) per formula unit of the energetically most favorable structures at calculated equilibrium volumes. The local optimizations were performed within the LDA-PZ, B3LYP, and GGA-PBE approximations.

**STRUCTURE TYPE**	**SPACE GROUP**	**B3LYP**	**GGA-PBE**	**LDA-PZ**
α-Y_2O_2S	*P*-3*m*1 (no. 164)	0	0	0
β-Y_2O_2S	$Cmc2_1$ (no. 36)	0.69	0.57	0.54
γ-Y_2O_2S	*Pm* (no. 6)	0.73	0.71	0.78
δ-Y_2O_2S	$Pmn2_1$ (no. 31)	0.96	0.83	0.85
ε-Y_2O_2S	$Cmc2_1$ (no. 36)	1.11	1.07	1.10
